# A New Edge Patch with Rotation Invariance for Object Detection and Pose Estimation

**DOI:** 10.3390/s20030887

**Published:** 2020-02-07

**Authors:** Xunwei Tong, Ruifeng Li, Lianzheng Ge, Lijun Zhao, Ke Wang

**Affiliations:** State Key Laboratory of Robotics and System, Harbin Institute of Technology, Harbin 150001, China; tong1137@163.com (X.T.); gelz@hit.edu.cn (L.G.); zhaolj@hit.edu.cn (L.Z.); wangke@hit.edu.cn (K.W.)

**Keywords:** object detection, object pose estimation, edge patch, rotation invariance

## Abstract

Local patch-based methods of object detection and pose estimation are promising. However, to the best of the authors’ knowledge, traditional red-green-blue and depth (RGB-D) patches contain scene interference (foreground occlusion and background clutter) and have little rotation invariance. To solve these problems, a new edge patch is proposed and experimented with in this study. The edge patch is a local sampling RGB-D patch centered at the edge pixel of the depth image. According to the normal direction of the depth edge, the edge patch is sampled along a canonical orientation, making it rotation invariant. Through a process of depth detection, scene interference is eliminated from the edge patch, which improves the robustness. The framework of the edge patch-based method is described, and the method was evaluated on three public datasets. Compared with existing methods, the proposed method achieved a higher average F1-score (0.956) on the Tejani dataset and a better average detection rate (62%) on the Occlusion dataset, even in situations of serious scene interference. These results showed that the proposed method has higher detection accuracy and stronger robustness.

## 1. Introduction

Object detection and pose estimation (ODPE) are important research topics in semantic navigation, robotic intelligent manipulation, and other fields. Although intensive work has been conducted, ODPE tasks remain challenging owing to scene interference problems. In this paper, only two kinds of scene interference, i.e., foreground occlusion and background clutter, are involved. In general, there are ODPE methods based on artificial features (local or global), machine learning, and local patches.

Global feature-based methods are robust to background clutter, but will suffer in situations with occlusion [1,2,3,4,5]. Local feature-based methods are robust to foreground occlusion, but only perform well for objects with enough feature points [6,7,8,9]. Furthermore, the representation ability of artificial features is not adequate for the diversity of objects.

Additionally, ODPE methods based on machine learning have achieved many remarkable results [10,11,12]. Compared with artificial feature-based methods, these learning-based methods are more adaptable to objects with various attributes. The object pose can be learned by random forests [13,14,15] or convolutional neural networks (CNNs) [16,17,18]. These methods directly use raw images for end-to-end learning and prediction, achieving real-time performance. However, the random forests or CNNs used in ODPE tasks need to be retrained for each new target object, which makes the learning-based methods not flexible enough.

Recently, local patch-based methods have been proposed, which use machine learning frameworks to learn adaptive descriptors of local red-green-blue and depth (RGB-D) patches. For instance, Doumanoglou et al. [19] trained a sparse auto-encoder to encode local RGB-D patches extracted from synthetic views and testing scenes. However, the scene interference contained in the patch reduces the matching accuracy between patches, leading to performance degradation during ODPE tasks. To improve the robustness of random forests against scene interference, Tejani et al. [20] integrated a z-check process into the similarity detection of training patches. However, without obviating the scene interference in the patches, the improvement in robustness brought by learning methods is limited. Kehl et al. [21] eliminated regions of scene interference in the depth channel by checking depth values, leaving RGB channels unconsidered.

Moreover, as far as the authors know, the traditional RGB-D patches have little rotation invariance, including those used by Kehl et al. [21]. This is because no canonical directions are selected, and the feature encoders are sensitive to the in-plane rotation of input data. To solve these problems, Zhang et al. [22] expanded the patch dataset by rotating the view of each rendering viewpoint at 10-degree intervals. However, this strategy introduces rotation quantization errors of up to 5 degrees (half of the rotation interval), which affect the accuracy of feature matching.

Therefore, an RGB-D patch with rotation invariance and robustness against scene interference is desired. For this reason, a new edge patch (E-patch) is proposed in this study. The E-patch is a local RGB-D patch centered at the edge pixel of the depth image. The advantages of the E-patch are summarized as follows:

- The E-patch is rotation invariant. In the sampling process, a canonical orientation is extracted to make the E-patch rotation invariant. Thus, it is not necessary to expand the E-patch library by rotating rendering views of the target object, avoiding quantization errors in the process of feature matching.
- The E-patch contains less scene interference. During the depth detection process, the scene interference is eliminated in the four channels of E-patch. This ensures the robustness of the E-patch against scene interference.

These two advantages result in the proposed E-patch-based method obtaining higher detection accuracy and stronger robustness to scene interference.

The rest of this paper is organized as follows: Section 2 describes the generation, encoding, and usage of E-patch. The experimental results and discussion are presented in Section 3, and Section 4 concludes the paper.

## 2. Methods

### 2.1. E-Patch Generation

#### 2.1.1. Sampling Center Extraction

**Figure 1:**
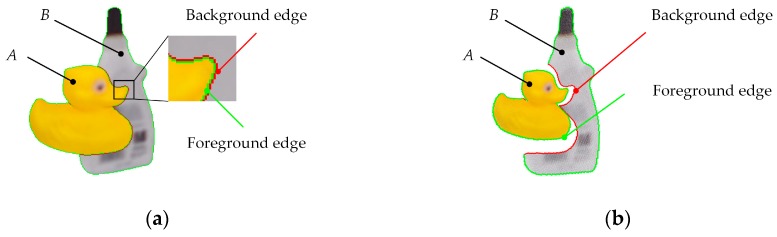
Schematic diagram of foreground edges (colored in green) and background edges (colored in red): (**a**) depth edges marked on the red-green-blue (RGB) image; (**b**) depth edges marked on the point cloud.

A schematic diagram of occlusion between object *A* (Duck) and object *B* (Glue) is shown in Figure 1.

**Figure 2:**
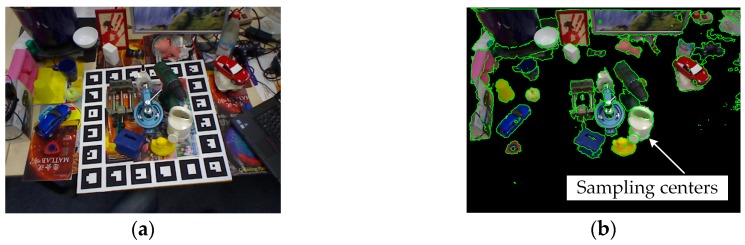
Illustration of sampling center extraction: (**a**) original image; (**b**) scene image overlaid with sampling centers (colored in green).

Using the gradient filtering algorithm, edges in the depth image were extracted and divided into foreground edges and background edges. These two kinds of depth edges are marked on the RGB image (Figure 1a) and point cloud (Figure 1b). Because the background edges could not represent the real contour of object *B*, only foreground edge pixels were selected as sampling centers. The selection criterion was defined by Equation (1):

(1) $$z_{edge}<\max\left(z_{neighbor}\right)-\delta_{edge}$$

where *z_edge_* is the depth value of the query edge pixel, *z_neighbor_* are the depth values of edge pixels in the 3 × 3 neighborhood of the query edge pixel, and *δ_edge_* is the threshold used in the abovementioned gradient filtering process. Figure 2 shows an extracting result. The desktop in Figure 2a was firstly extracted using the random sampling consensus (RANSAC) algorithm [23], and irrelevant scene points (black pixels in Figure 2b) under the desktop were removed. Sampling centers are drawn as green pixels in Figure 2b.

#### 2.1.2. E-Patch Sampling along a Canonical Orientation

**Figure 3:**
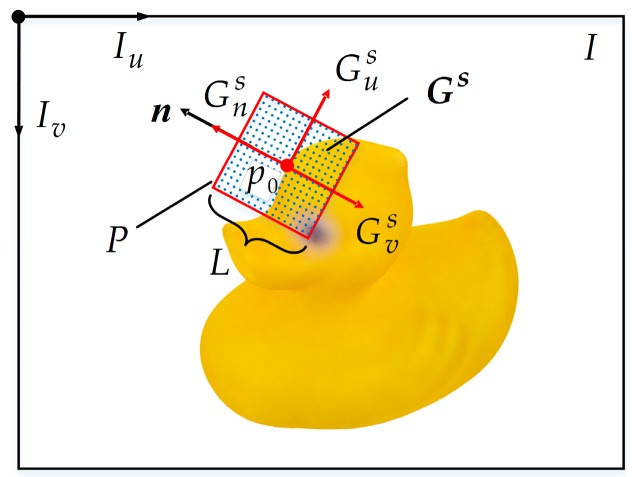
Illustration of the sampling process of the E-patch.

The sampling process of E-patch is shown in Figure 3. An E-patch *P* with a size of 32 × 32 × 4 was sampled from a square region in the input image *I*. The image coordinate frame of *I* is ${Frame}_{I}$, which has the principal axes $I_{u}$ and $I_{v}$. The sampling square’s coordinate frame ${Frame}_{s}$ is marked with its principal axes ($G_{u}^{s}$, $G_{v}^{s}$). $G_{n}^{s}$ is the canonical orientation of ${Frame}_{s}$.

The sampling square is centered at the edge pixel *p*_0_ and has a side length of *L*. To make the E-patch scale invariant, *L* was calculated via Equation (2):

(2) $$L=〚\frac{L_{s}}{z_{0}}\cdot f_{c}〛,$$

where $L_{s}$ = 50 mm is a fixed metric size of the E-patch, $f_{c}$ is the focal length of the camera, $z_{0}$ is the depth of *p*_0_, and 〚 〛 is the rounding function.

Each neighboring edge pixel of *p*_0_ within the distance of *L/2* was collected and denoted as $p_{i}$ (*i* = 1, 2, …). To make the E-patch rotation invariant, the canonical orientation $G_{n}^{s}$ of the sampling square was aligned with a unit vector ***n***, which was determined by Equation (3):

(3) $$n=\frac{g}{\left\|g\right\|},$$

where the weighted sum ***g*** of gradient directions was calculated using Equation (4):

(4) $$g=\sum_{i}w_{i}g_{i}.$$

In Equation (4), $g_{i}$ is the gradient direction of $p_{i}$, and the weighting coefficient $w_{i}$ was calculated by Equation (5):

(5) $$w_{i}=e^{-36d_{i}/L^{2}},$$

where $d_{i}$ is the pixel distance between *p*_0_ and $p_{i}$.

During the sampling process, a point set $G^{s}=\{G_{\mathrm{ij}}\},\forall i,j\in \left\{1,\ldots ,32\right\}$, was arranged in the sampling square. The coordinates of *G_ij_* was calculated using Equation (6):

(6) $$\left(\begin{array}{c} u_{ij}^{I}\\ v_{ij}^{I} \end{array}\right)=R\left(\begin{array}{c} u_{ij}^{S}\\ v_{ij}^{S} \end{array}\right)+\left(\begin{array}{c} u_{0}\\ v_{0} \end{array}\right),\;\forall i,j\in \left\{1,\ldots ,32\right\},$$

where ($u_{ij}^{I}{,v}_{ij}^{I}$) and ($u_{ij}^{S}{,v}_{ij}^{S}$) are the coordinates of $G_{ij}$ in ${Frame}_{I}$ and ${Frame}_{s}$, respectively, and ($u_{0},v_{0}$) is the coordinate of *p*_0_ in ${Frame}_{I}$. $u_{ij}^{S}$ and $v_{ij}^{S}$ were respectively calculated by Equations (7) and (8):

(7) $$u_{i\cdot }^{S}=-\frac{L}{2}+\frac{i-1}{31}L,$$

(8) $$v_{\cdot j}^{S}=-\frac{L}{2}+\frac{j-1}{31}L.$$

The rotation matrix ***R*** was expressed as Equation (9):

(9) $$R=\left(\begin{array}{cc} -n_{v} & -n_{u}\\ n_{u} & -n_{v} \end{array}\right),$$

where $n_{u}$ and $n_{v}$ are the horizontal and vertical components of ***n*** in ${Frame}_{I}$.

As described in Equation (10), the E-patch *P* was obtained by sampling the original image *I* in four RGB-D channels using the same rules:

(10) $$P\left(i,j,c\right)=I\left(u_{ij}^{I},v_{ij}^{I},c\right),\;\forall i,j\in \left\{1,\ldots ,32\right\},\;\forall c\in \left\{red,green,blue,depth\right\}.$$

In the E-patch, the pixel values in RGB channels ranged from 0 to 255, while in the depth channel, values ranged from 0 mm to 4000 mm. To balance pixel values in the four channels, Equations (11) and (12) were applied to each E-patch:

(11) $${P^{\prime }}_{depth}=\frac{P_{depth}-z_{0}}{3\times L_{s}},$$

(12) $${P^{\prime }}_{rgb}=\frac{P_{rgb}}{128}-1,$$

where *P_depth_* are pixel values in the depth channel, *P_rgb_* are pixel values in RGB channels, and $P_{depth}^{\prime }$ and $P_{rgb}^{\prime }$ are the corresponding updated pixel values.

#### 2.1.3. Depth Detection

**Figure 4:**
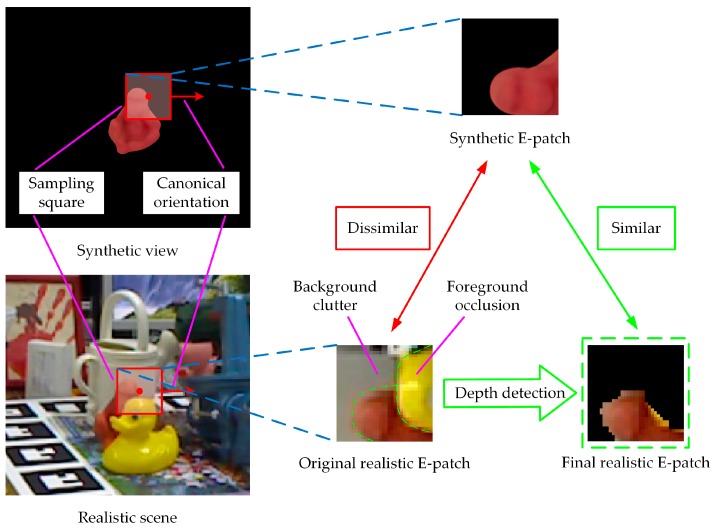
Illustration of the depth-detection process.

The key to ODPE methods based on the E-patch is the similarity matching between E-patches extracted from synthetic views and real scenes. The original E-patch in a realistic scene contains regions of foreground occlusion and background clutter, as shown in Figure 4. This leads to a difference between realistic and synthetic E-patches. Therefore, a process of depth detection was used to eliminate the regions of occlusion and clutter. Firstly, the regions of foreground occlusion were detected with a criterion of $P_{depth}^{\prime }<-1$, and patches with occlusive rates higher than 30% were abandoned. Then, the criterion of $P_{depth}^{\prime }>1$ was used to detect the regions of background clutter. All four channels were set to zero for pixels in the regions of occlusion and clutter, which enhanced the robustness of E-patch against scene interference.

### 2.2. Encoding Network Training

**Figure 5:**
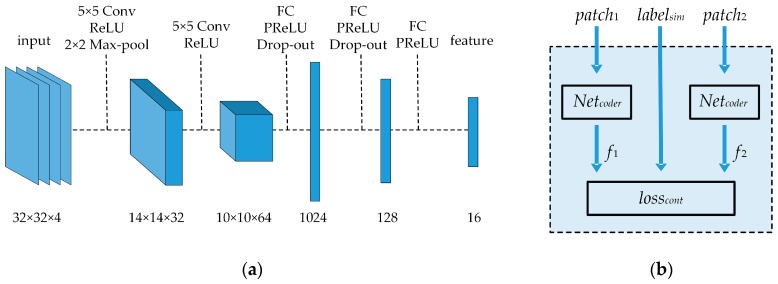
(**a**) Architecture of the feature-encoding network; (**b**) architecture of the Siamese network.

A CNN-based encoder, *Net_coder_*, was constructed and trained within the Siamese network framework. The *Net_coder_* takes in an E-patch and computes a 16-dimensional descriptor, as shown in Figure 5a. It included two convolutional layers (Conv) and three fully connected layers (FC). Each convolutional layer was followed by a rectified linear unit (ReLU) as the activation function. Since the size of the input E-patch was only 32 × 32 × 4, to avoid information loss, only one maximum pooling layer (Max-pool) was introduced after the first convolutional layer. Each fully connected layer was followed by a parametrized rectified linear unit (PReLU) as the activation function, which avoided the premature failure of neurons. Following the first two fully connected layers, there were dropout layers (Drop-out) to prevent overfitting of the training process.

Two parameter-sharing encoders *Net_coder_* were combined into a Siamese network, as shown in Figure 5b. *patch*_1_ and *patch*_2_ are E-patches in the patch pair, and *label_sim_* is the similarity label of the patch pair (*label_sim_* = 1 for a similar patch pair and *label_sim_* = 0 for a dissimilar pair). *f*_1_ and *f*_2_ are features of *patch*_1_ and *patch*_2_, respectively. The contrastive loss function *loss_cont_* is formalized in Equation (13):

(13) $$loss_{cont}=\frac{1}{2N}\sum_{i=1}^{N}\left[label_{sim}\cdot df_{i}^{2}+\left(1-label_{sim}\right)\cdot \max{\left(margin-df_{i},0\right)}^{2}\right],$$

where *N* is the number of patch pairs, *df_i_* is the Euclidean distance between features of the E-patches in the *i*th pair, and *margin* is the threshold value (which here was 1).

Patch pairs numbering 0.6 million were determined using the LineMod dataset [4] to train the Siamese network. The ratio of similar and dissimilar patch pairs was 1:1. The parameters of *Net_coder_* were optimized using the root-mean-square prop (RMSprop) algorithm to minimize the contrast loss *loss_cont_*. This was equivalent to gathering similar E-patches and alienating dissimilar ones.

### 2.3. Object Detection and Pose Estimation Based on E-patch

**Figure 6:**
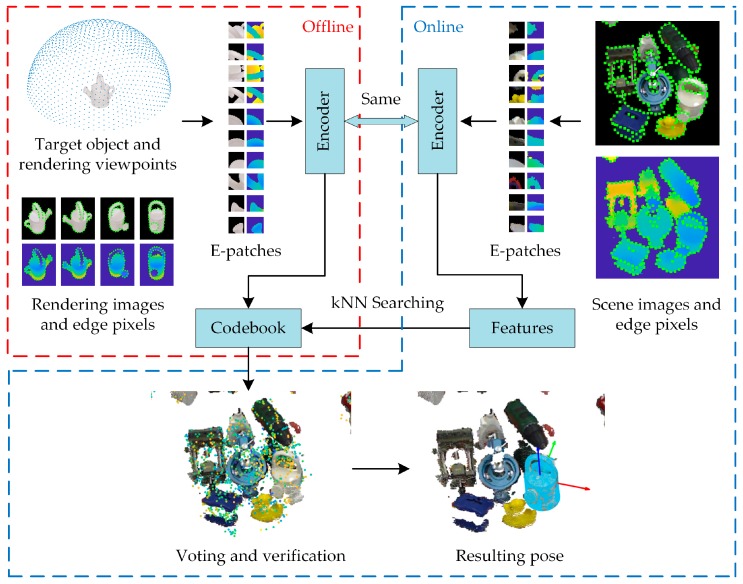
Framework of the E-patch-based method for object detection and pose estimation (ODPE) tasks.

The proposed E-patch-based method consists of two phases, offline modeling and online testing, as shown in Figure 6. In the online testing phase, processes of object detection and pose estimation were carried out simultaneously. The same CNN-based encoder was used in both phases to guarantee the consistency of the feature coding principle.

#### 2.3.1. Offline Construction of the Codebook

In the offline modeling phase, each target object was uniformly rendered from 1313 perspectives. Note that because of the rotation invariance of the E-patch, no in-plane rotation was needed for rendering views. Features of all E-patches in rendering images were computed and used to construct the codebook. To improve the retrieval efficiency, the codebook was arranged in a *k*-d tree according to Euclidean distances between features, which was denoted as ${Tree}_{F}$.

**Figure 7:**
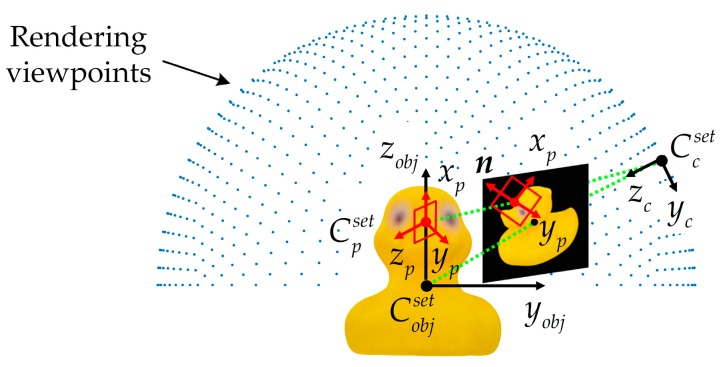
Coordinate systems used in the construction of the codebook.

All the coordinate systems used in the construction of the codebook are shown in Figure 7. $C_{obj}^{set}$, $C_{p}^{set}$, and $C_{c}^{set}$ are the local coordinate systems of the target object, synthetic E-patch, and rendering camera, respectively.

In the codebook, the feature of each E-patch was stored together with an annotation, *info* = {*obj*, *^set^*$T_{p}^{o}$}. Here, *obj* is the name of the target object, and *^set^*$T_{p}^{o}$ is the transformation from $C_{obj}^{set}$ to $C_{p}^{set}$, which was obtained by Equation (14):

(14) Tposet=Tcoset⋅Tpcset,

where Tcoset is a known transformation from $C_{obj}^{set}$ to $C_{c}^{set}$ and Tpcset is the transformation from $C_{c}^{set}$ to $C_{p}^{set}$, which was calculated via Equation (15):

(15) Tpcset=[−nv−nu0pxnu−nv0py001pz0001],

where (*p_x_*, *p_y_*, *p_z_*) is the spatial coordinate of the sampling center of the E-patch.

#### 2.3.2. Online Testing

In the online testing phase, the local coordinate systems of the target object, realistic E-patch, and testing camera were respectively denoted as $C_{obj}^{scene}$, $C_{p}^{scene}$, and $C_{c}^{scene}$. Then, the transformation relationship between coordinate systems in the scene is expressed as Equation (16):

(16) Tposcene=Tcoscene⋅Tpcscene,

where Tposcene is the transformation from $C_{obj}^{scene}$ to $C_{p}^{scene}$ and Tcoscene is the transformation from $C_{obj}^{scene}$ to $C_{c}^{scene}$, i.e., the pose of the target object. Tpcscene is the transformation from $C_{c}^{scene}$ to $C_{p}^{scene}$, which was also determined by the canonical orientation and sampling center of the scene E-patch, similarly to Equation (15).

For matching E-patches, it is reasonable to assume that the transformation relationship between the coordinate systems of the E-patch and the object in the realistic scene is the same as that in the virtual scene (i.e., Tposcene = Tposet). Therefore, according to Equation (14) and Equation (16), the object pose Tocscene was determined by Equation (17):

(17) Tocscene=(Tcoscene)−1=Tpcscene⋅(Tposet)−1.

Each E-patch in the testing scene was encoded as a feature *f* with the same encoder *Net_coder_* used in the offline phase. Its 100 nearest neighbors in *Tree_F_* were searched and denoted as *f_j_* (*j* = 1, …, 100). Each neighboring feature *f_j_* generated a vote *v_j_* = {*obj_j_*, Tojcscene} based on its annotation *info_j_* = {*obj_j_*, Tpjoset} stored in the codebook. The confidence *conf_j_* of vote *v_j_* was calculated by Equation (18):

(18) $$conf_{j}=w_{j}\cdot \alpha_{j},$$

where weighting coefficients $w_{j}$ and $\alpha_{j}$ are respectively calculated according to Equations (19) and (20):

(19) $$w_{j}=e^{-\left\|f-f_{j}\right\|},$$

(20) $$\alpha_{j}=w_{j}/\sum_{j=1}^{100}w_{j}.$$

The mean shift algorithm was used to cluster voting poses successively in the translational space and rotational space. For each cluster of votes, the clustering center was regarded as a hypothetical pose, and the total weight was regarded as the corresponding confidence. To ensure operational efficiency, only the top 80% of hypothetical poses according to their confidence values were retained. After a hypothesis verification process similar to that used by Li et al. [7], the estimated results were finally achieved.

## 3. Experiments and Discussion

In this section, the robustness of our E-patch-based method to occlusion and clutter is demonstrated through two experiments on public datasets. The results of these two experiments also show the improvement in the detection accuracy. In addition, experimental results on the third dataset indicate that our method also has high accuracy in the case of slight clutter.

### 3.1. Results on the Tejani Dataset

#### 3.1.1. Detection Results

**Figure 8:**
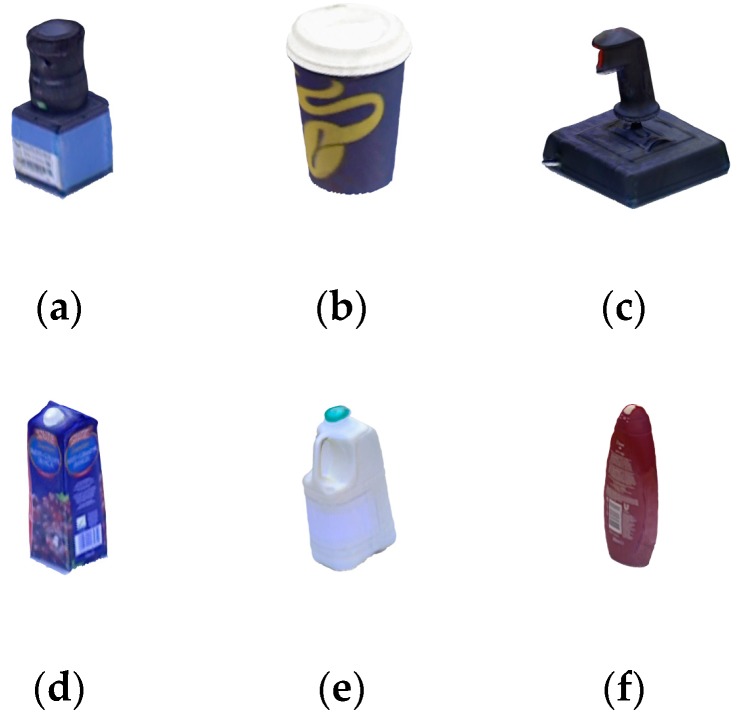
Six objects in the Tejani dataset: (**a**) Camera; (**b**) Coffee Cup; (**c**) Joystick; (**d**) Juice Carton; (**e**) Milk; (**f**) Shampoo.

The Tejani dataset [20] was chosen to demonstrate the robustness of the proposed method to background clutter, which contains six target objects as shown in Figure 8. The numbers of testing scenes contained in each object are 337, 501, 838, 556, 288, and 604, respectively. In each testing image, there are two or three instances of the same kind of target object. Although this dataset contains slight occlusion, different levels of background clutter pose a challenge to ODPE tasks.

**Figure 9:**
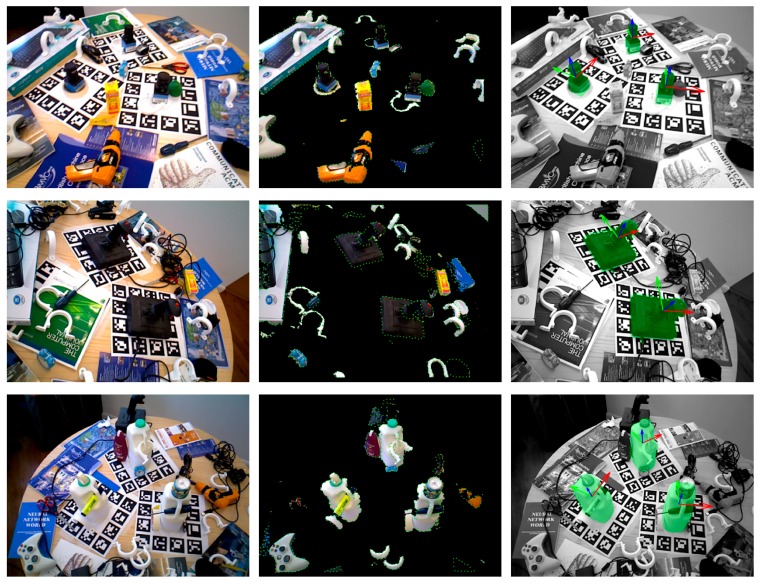
Some detection results on the Tejani dataset.

Figure 9 shows the results of our method in three testing scenes. In each row, the left subfigure is a scene image, the middle subfigure is a preprocessed scene overlaid with edge pixels, and estimated poses are shown in the right subfigure with green transparent models, where the scene is displayed in gray for better visibility.

An estimated pose was considered correct when its intersection over union (IoU) score was higher than 0.5 [16]. The F1-scores of the proposed method are compared with those of the state-of-the-art methods in Table 1. The results of the comparison methods were obtained from [7,24]. The proposed method obtained a higher average F1-score (0.956) than did the other methods (0.910, 0.885, 0.747, and 0.939). This indicates that the use of the E-patch provided higher detection accuracy.

The method in [7] depends only on depth information. Due to the small size of ‘Camera’, its space points are insufficient, resulting in a significant reduction in F1-score. The methods in [16,21] are learning-based methods trained with synthetic models. Therefore, differences between synthetic and realistic scenes caused by the scene interference affect the detection results. This is especially true for the small object ‘Camera’ and pure white object ‘Milk’.

**Figure 10:**
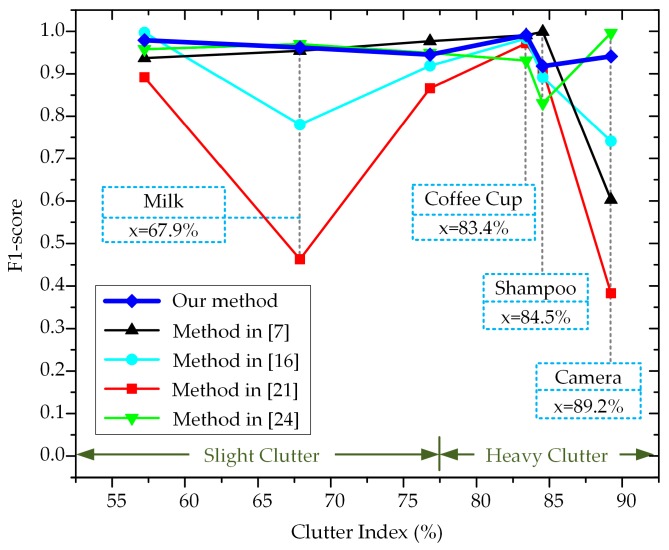
F1-scores against different levels of background clutter.

For each object, a clutter index was designed to represent the clutter level quantitatively. It was calculated as the average proportion of background region in a radial neighborhood of 50 pixels (around the projection of the object center). The clutter index of each object is shown in Table 2. Taking the clutter index as the abscissa axis and the F1-score as the ordinate axis, curves were drawn and are presented in Figure 10 to illustrate the influence of background clutter on the F1-scores of all mentioned methods.

Taking a clutter index of 77.5% as the dividing point, objects were divided into two groups: those with slight clutter (‘Joystick’, ‘Milk’, and ‘Juice Carton’) and those with heavy clutter (‘Coffee Cup’, ‘Shampoo’, and ‘Camera’). For the objects with slight clutter, the average F1-score of the proposed method was 0.962, while those of the methods in [7,16,21,24] were 0.956, 0.899, 0.74, and 0.959, respectively. The pure white color of ‘Milk’ made RGB-D patches inside the object too similar to distinguish, leading to failure in [21]. The E-patch was located at the depth edge and contained features of object contours as well as RGB-D appearance. Moreover, by sampling along the canonical orientation, descriptor variation caused by in-plane rotation was avoided.

For the objects with heavy clutter, our average F1-score was 0.95, while those of the methods in [7,16,21,24] were 0.864, 0.872, 0.755, and 0.919, respectively. With the aggravation of background clutter, our average F1-score decreased by 0.012, while those of the methods in [7,16,24] decreased by at least 0.027. These data prove that the proposed E-patch achieved stronger robustness against clutter. The reasons for these phenomena are explained in detail later. Note that the average F1-score of the method in [21] increased by 0.015 because its poor performance on the object ‘Milk’, which made this method unsuitable for robustness analysis.

The aforementioned improvement in detection accuracy and robustness against clutter are owing to the advantages of the E-patch. Considering a synthetic E-patch *P_syn_* and the corresponding realistic E-patch *P_rel_*, the relationship between them is expressed as Equation (21):

(21) $$P_{rel}=P_{syn}+\frac{\partial P}{\partial \theta }\cdot d\theta +\varepsilon ,$$

where *ε* is variation of the E-patch caused by background clutter, and *dθ* is the deviation angle of the in-plane rotation.

Taking *E*(·) as the encoding function, the features of the two E-patches are obtained via Equations (22) and (23):

(22) $$f_{syn}=E\left(P_{syn}\right),$$

(23) $$f_{rel}=f_{syn}+\frac{\partial E}{\partial P}\cdot \left(\frac{\partial P}{\partial \theta }\cdot d\theta +\varepsilon \right),$$

where *f_syn_* and *f_rel_* are the features of *P_syn_* and *P_rel_*, respectively. Therefore, the feature distance between *f_syn_* and *f_rel_* can be expressed as Equation (24):

(24) $$dis=\left\|\frac{\partial E}{\partial P}\right\|\cdot \left\|\frac{\partial P}{\partial \theta }\cdot d\theta +\varepsilon \right\|.$$

The rotation invariance of the E-patch made *dθ* ≈ 0 (‘≈’ indicates ‘close to’), and the elimination of background clutter in the depth detection process made *ε* ≈ 0. Both of these led to *dis* ≈ 0, and a smaller *dis* means a more accurate feature-matching result. Consequently, the E-patch is beneficial to improving the detection accuracy and robustness to clutter of ODPE methods.

#### 3.1.2. Computation Time

The average time of our online testing phase on the Tejani dataset was 903.4 ms, which is close to the 774.5 ms in Liu et al. [24]. The online testing phase consisted of four stages, namely, ‘Patch sampling’, ‘Feature encoding’, ‘Hypothesis generation’, and ‘Hypothesis verification’. The ‘Feature encoding’ stage was implemented in a Jupyter notebook environment with an NVIDIA Tesla T4 graphics processing unit (GPU). Other stages were implemented in a MATLAB environment, running on a laptop with an Intel central processing unit (CPU, i7-4720HQ).

In our online testing phase, the computation times of each stage were 153.9 ms, 16.5 ms, 228.7 ms, and 504.3 ms, respectively, while those in [24] were 12.5 ms, 47.4 ms, 186.2 ms, and 528.4 ms, respectively. In ‘Feature encoding’ and ‘Hypothesis verification’, our times were roughly the same as those in [24]. The introduction of a canonical orientation led to longer times in ‘Patch sampling’ and ‘Hypothesis generation’, which was acceptable considering the improvement in the detection accuracy. In addition, the computation time of the depth detection process was negligible.

### 3.2. Results on the Occlusion Dataset

**Figure 11:**
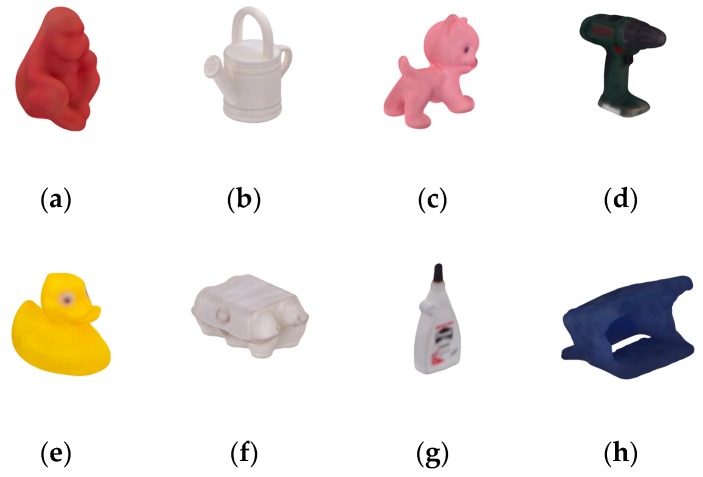
Eight objects in the Occlusion dataset: (**a**) Ape; (**b**) Can; (**c**) Cat; (**d**) Driller; (**e**) Duck; (**f**) Egg Box; (**g**) Glue; (**h**) Hole Punch.

The Occlusion dataset [14] was used to test the robustness of the proposed method to the occlusion problem. Figure 11 shows the eight objects in the dataset. To compare with the testing results reported in [25], the same 200 scenes were selected. All eight objects coexist and occlude each other in each testing scene, which is challenging for ODPE tasks.

**Figure 12:**
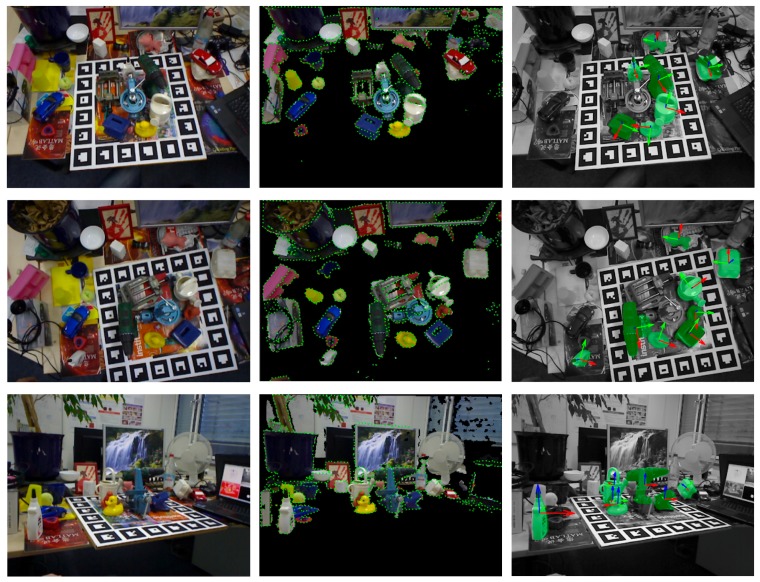
Some detection results on the Occlusion dataset.

Detection results of our method in three scenes are shown in Figure 12. In each row, the left subfigure is a scene image, the middle subfigure is a preprocessed scene overlaid with edge pixels, and the right subfigure shows estimated poses with green transparent models, where the scene is displayed in gray for better visibility.

Any estimated pose with a visible surface discrepancy (VSD) score of less than 0.3 was considered correct [25]. The detection rates (percentages of correct poses) of all the eight objects in the Occlusion dataset were calculated, as shown in Table 3.

The results of the comparison methods were taken from [25]. Our method increased the detection rates of ‘Cat’, ‘Driller’, ‘Glue’, and ‘Hole Punch’ by 1%, 10%, 11%, and 6%, respectively. Our average detection rate (62%) was higher than those of other methods (51%, 58%, 54%, and 51%), which showed that our E-patch-based method had higher accuracy.

The methods used in [8,9] rely only on the point pair feature, and perform well in most scenes with good point-cloud quality. However, when the main plane of a flat object (‘Glue’) flips, its point cloud quality deteriorates rapidly. This leads to a significant reduction in the detection rate.

**Figure 13:**
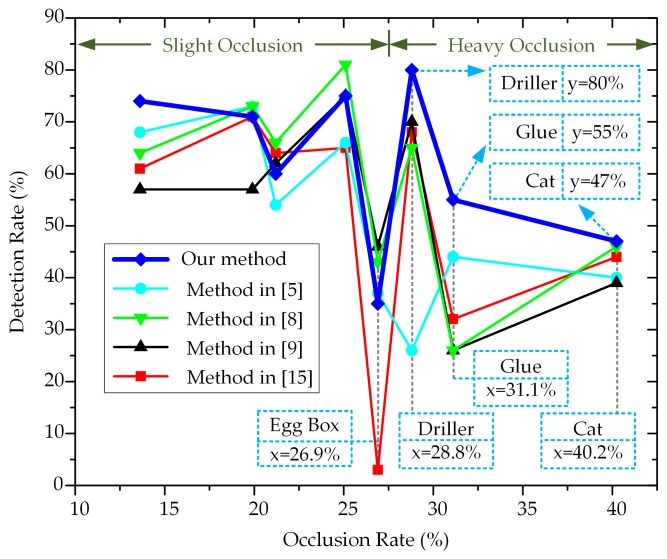
Detection rates against different levels of occlusion.

For quantitative analysis, Table 4 shows the occlusion rate of each object, which is the average proportion of occlusion in all testing scenes. The detection rates of all five methods against different occlusion rates are displayed in Figure 13.

Using an occlusion rate of 27.5% as the boundary, the objects were divided into two groups: those with slight occlusion (‘Hole Punch’, ‘Duck’, ‘Ape’, ‘Can’, and ‘Egg Box’) and those with heavy occlusion (‘Driller’, ‘Glue’, and ‘Cat’). For the objects in the first group, our average detection rate was 63%, while those of the methods in [5,8,9,15] were 59.6%, 65.4%, 59.4%, and 52.8%, respectively. This means that the E-patch has acceptable performance in the situation of slight occlusion (only lower than the method in [8]). In particular, ‘Egg Box’ was the most difficult object for the proposed method, because its textureless appearance and repeated edges made E-patches too similar to distinguish. This problem may be solved by introducing a sophisticated process of hypothesis verification, which will be conducted in future work.

For the objects in the second group, our average detection rate was 60.7%, while those of the methods in [5,8,9,15] were 36.7%, 45.7%, 45%, and 48%, respectively. Owing to the heavy occlusion, our average detection rate decreased by 2.3%, which was a lower decrease than those for the aforementioned methods (decreased by at least 4.8%). These results indicate that the E-patch is more robust to occlusion problems. They also prove the effectiveness of eliminating occlusion regions during the depth detection process.

Similar to the theoretical analysis of the first experiment, our improvement in detection accuracy and robustness to occlusion can be explained by Equation (25):

(25) $$dis=\left\|\frac{\partial E}{\partial P}\right\|\cdot \left\|\frac{\partial P}{\partial \theta }\cdot d\theta +\varepsilon^{\prime }\right\|,$$

where *ε*’ represents the alteration of the E-patch caused by foreground occlusion.

The rotation invariance of E-patch made *dθ* ≈ 0, and the elimination of occlusion regions in the depth detection process made *ε*’ ≈ 0. Therefore, *dis* ≈ 0, and feature matching became more accurate. Consequently, E-patch is conducive to increased detection accuracy and robustness to occlusion in ODPE methods.

### 3.3. Results on the Doumanoglou Dataset

**Figure 14:**
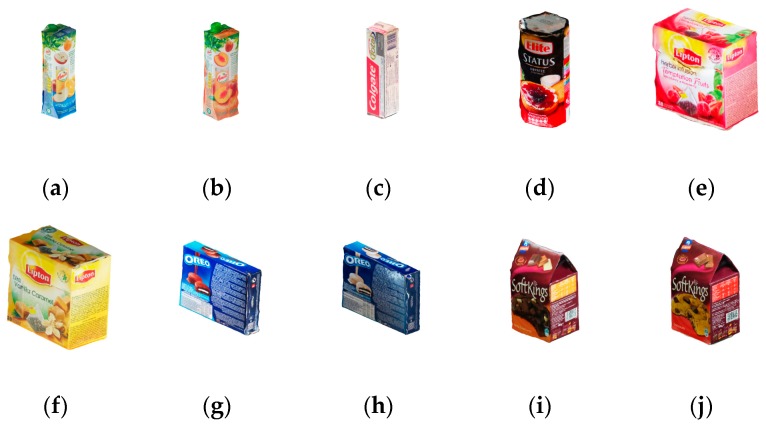
Ten objects in the Doumanoglou dataset: (**a**) Amita–1; (**b**) Amita–2; (**c**) Colgate; (**d**) Elite; (**e**) Lipton–1; (**f**) Lipton–2; (**g**) Oreo–1; (**h**) Oreo–2; (**i**) Soft Kings–1; (**j**) Soft Kings–2.

The Douanoglou dataset [19] was chosen to demonstrate the effectiveness of the proposed E-patch-based method in the case of light clutter. Figure 14 shows the 10 objects in the dataset, four pairs of which belong to the same category.

**Figure 15:**
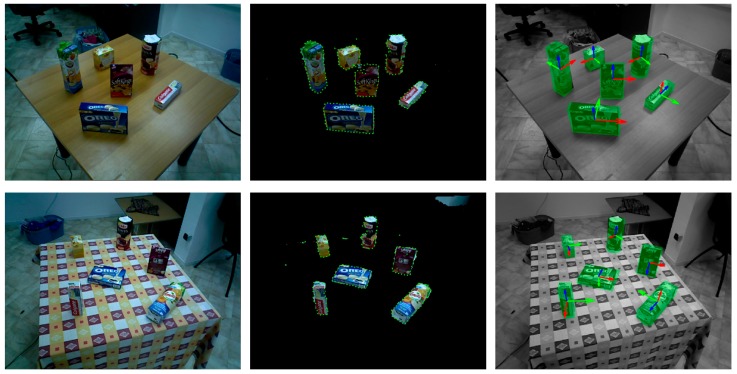
Some detection results on the Doumanoglou dataset.

Compared with the first two datasets, the Douanoglou dataset contains less background clutter, which is suitable for analyzing the basic detection performance of ODPE methods. The dataset contains 351 testing scenes, each of which has multiple objects placed on the desktop. The detection results of our method in two scenes are shown in Figure 15.

In each row, the left subfigure is a scene image, the middle subfigure is a preprocessed scene overlaid with edge pixels, and estimated poses are shown in the right subfigure with green transparent models, where the scene is displayed in gray for better visibility.

The clutter index of each object is shown in Table 5, which indicates the Doumanoglou dataset has slight clutter.

An estimated pose was considered correct when its IoU score was higher than 0.5. As shown in Table 6, our detection rates were generally higher than those of the method in [19], which revealed the high accuracy of the proposed method in the case of slight clutter. It should be noted that the method in [19] has a low detection rate for the ‘Colgate’ object. This is because the narrow surfaces of ‘Colgate’ result in too many RGB-D patches near the edge. These patches usually contain background clutter, which cannot be eliminated by the method in [19]. ‘Lipton’ and ‘Oreo’ have similar problems.

## 4. Conclusions

A new E-patch for ODPE tasks was proposed herein. The advantages of the E-patch were described and evaluated on three public datasets. The proposed method improved the F1-score from 0.939 to 0.956 on the Tejani dataset and improved the detection rate from 58% to 62% on the Occlusion dataset. With intensifying background clutter, the F1-score of the proposed method decreased more slightly (0.012) than did those of the comparison methods (more than 0.027). When the occlusion level increased, the detection rate of the proposed method decreased by 2.3%, and those of the comparison methods decreased by at least 4.8%. These results prove that the proposed method is more accurate and robust to scene interference. Additionally, one limitation of the proposed method is that it fails to cover texture-less objects with repeated edges, which is worth further study.

## Figures and Tables

**Table 1:** F1-scores of methods on the Tejani dataset.

Objects	Li et at. [7]	Kehl et al. [16]	Kehl et al. [21]	Liu et at. [24]	Ours
Camera	0.603	0.741	0.383	0.996	0.941
Coffee Cup	0.991	0.983	0.972	0.931	0.990
Joystick	0.937	0.997	0.892	0.958	0.979
Juice Carton	0.977	0.919	0.866	0.949	0.945
Milk	0.954	0.780	0.463	0.970	0.962
Shampoo	0.999	0.892	0.910	0.831	0.918
Average	0.910	0.885	0.747	0.939	0.956

**Table 2:** Clutter index of each object in the Tejani dataset (%).

Objects	Clutter Indexes
Joystick	57.2
Milk	67.9
Juice Carton	76.8
Coffee Cup	83.4
Shampoo	84.5
Camera	89.2

**Table 3:** Detection rates of methods on the Occlusion dataset (%).

Objects	Hodan et al. [5]	Vidal et al. [8]	Drost et al. [9]	Brachmann et al. [15]	Ours
Ape	54	66	62	64	60
Can	66	81	75	65	75
Cat	40	46	39	44	47
Driller	26	65	70	68	80
Duck	73	73	57	71	71
Egg Box	37	43	46	3	35
Glue	44	26	26	32	55
Hole Punch	68	64	57	61	74
Average	51	58	54	51	62

**Table 4:** Occlusion rate of each object in the Occlusion dataset (%).

Objects	Occlusion Rates
Hole Punch	13.6
Duck	19.9
Ape	21.2
Can	25.1
Egg Box	26.9
Driller	28.8
Glue	31.1
Cat	40.2

**Table 5:** Clutter index of each object in the Doumanoglou dataset (%).

Objects	Clutter Indexes
Amita	13.6
Colgate	55.5
Elite	8.3
Lipton	56.1
Oreo	22.3
Soft Kings	25.5

**Table 6:** Detection rates of methods on the Doumanoglou dataset (%).

Objects	Doumanoglou et at. [19]	Ours
Amita	71.2	72.5
Colgate	28.6	53.6
Elite	77.6	85.7
Lipton	59.2	78.6
Oreo	59.3	87.5
Soft Kings	75.9	91.1
Average	62.0	78.2
